# Health-economic evaluation of the outpatient, inpatient, and public health sector in Germany: Insights from the first three COVID-19 waves

**DOI:** 10.1371/journal.pone.0314164

**Published:** 2025-02-07

**Authors:** Afschin Gandjour

**Affiliations:** Frankfurt School of Finance & Management, Frankfurt am Main, Germany; University of Hong Kong, HONG KONG

## Abstract

**Aim:**

The aim of this study is to quantify the effectiveness and cost-effectiveness of the outpatient and inpatient sectors (specifically intensive care units, ICUs) and local health departments in managing the first three waves of the COVID-19 pandemic in Germany.

**Methods:**

The analysis is based on a modelling approach using secondary data. The effectiveness of each sector was measured by determining the reduction in the case fatality rate (CFR) of COVID-19 patients by May 7, 2021. A counterfactual scenario assuming the absence of each sector was used to quantify their effectiveness. Direct medical costs for each sector were calculated from a statutory health insurance perspective, utilizing reimbursement rates for both the inpatient and outpatient sectors. Incremental cost-effectiveness ratios (ICERs) were determined, representing the costs per death avoided.

**Results:**

The ICUs achieved the greatest reduction in the CFR of COVID-19 patients during the first three waves (1.9%). The outpatient sector followed with a reduction of 1.4%, and the local health departments contributed to a 0.3% decrease in the CFR. In terms of spending, ICUs had the highest expenditures among the sectors, resulting in an ICER of €59,055 per death avoided. On the other hand, local health departments were costlier but less effective than the outpatient sector. Results remained consistent across various input assumptions.

**Conclusion:**

During the first three waves of the COVID-19 pandemic in Germany, the inpatient sector (ICUs) made the largest contribution to preventing deaths while also incurring the highest costs.

## Introduction

Most cost-effectiveness analyses on COVID-19 have focused on evaluating specific pharmacological and non-pharmacological interventions. These interventions encompass a range of strategies including personal protective equipment, testing, contact tracing, isolation, quarantine, social distancing, closure of schools and universities, vaccinations against COVID-19, and treatments of COVID-19 [1]. However, there remains a dearth of research on the cost-effectiveness of different levels of health care delivery for treating COVID-19, particularly concerning the primary, secondary and tertiary levels of health care.

The German healthcare system operates within an inpatient and outpatient sector [2]. All treatments and services provided outside hospitals are classified under outpatient care [2], with hospitals occasionally offering outpatient care through specialist outpatient departments [2]. However, treatments combining inpatient and outpatient care are relatively uncommon [2]. An intriguing aspect the German healthcare system is that, by law, outpatient care is given priority over inpatient care (Social Code Book (Sozialgesetzbuch, SGB) XII §13 section 1).

During the first wave of the COVID-19 pandemic in Germany, a debate arose between the German Hospital Association (DKG) and the National Association of Statutory Health Insurance Physicians (Kassenärztliche Bundesvereinigung, KBV) regarding which sector (outpatient or inpatient) played a larger role in coping with the crisis [3]. The KBV claimed that 6 out of 7 COVID-19 patients received outpatient care, effectively mitigating the burden on the inpatient sector and preventing COVID-19 outbreaks in hospitals [4]. Building on the contribution of the outpatient sector, the KBV [4] argued for maintaining the decentralized organization of outpatient care in Germany.

Similarly, the importance of local health departments in managing the COVID-19 pandemic has been emphasized in Germany. The primary responsibilities of local health departments include breaking chains of infection, implementing and monitoring quarantine measures, and tracing contacts. Former German Chancellor Angela Merkel acknowledged the important role played by local health departments in the fight against the pandemic [5].

The present study aims to quantify the effectiveness and cost-effectiveness of the outpatient and inpatient sectors, along with the local health departments, in managing the first three waves of the COVID-19 pandemic in Germany. By assessing the impact and costs of each sector, this research will contribute to a retrospective understanding of the optimal allocation of healthcare resources during the pandemic. Moreover, the findings will inform future pandemic response planning and resource allocation.

## Methods

### Conceptual approach

The study adopts an analytical model, specifically a mathematical model with a closed-form solution. The model utilizes secondary data for analysis. All methods were carried out in accordance with relevant guidelines and regulations. No experimental protocols were employed in this study as it did not involve experimental research.

The primary outcome is the reduction in the case fatality rate (CFR) of COVID-19 patients. Measuring the mortality of COVID-19 patients is a key patient-relevant endpoint in evaluating the impact of pandemic management strategies. The effectiveness of each sector is quantified by comparing actual outcomes to a counterfactual scenario assuming the absence of that sector. This approach was chosen due to the lack of a randomized controlled trial allocating COVID-19 patients to the outpatient or inpatient sector, or a trial randomly assigning contacts of COVID-19 patients to contact tracing or no contact tracing.

Effectiveness in the inpatient sector (ICUs) is measured by the reduction in the CFR due to intensive care treatment. Key parameters include the probability of ICU treatment, additional mortality rate without ICU treatment, and the false positive rate of ICU admissions. The outpatient sector’s effectiveness is assessed through a counterfactual scenario where all patients are treated as inpatients, increasing the transmission rate and associated mortality. Effectiveness in local health departments is evaluated based on the reduction in the death toll achieved through contact tracing and quarantine measures.

The study is conducted from the perspective of the statutory health insurance, focusing on direct medical costs. Cost data for the inpatient sector include ICU operating expenses derived from diagnosis-related group (DRG) codes and additional tariffs. Costs for the outpatient sector are calculated based on the outpatient fee schedule of the statutory health insurance. Costs for local health departments encompass labor and PCR testing for contact tracing.

The analysis covers the period of the first three COVID-19 waves in Germany, ending on May 7, 2021.

### Effectiveness

The effectiveness was modelled using available secondary data. In the inpatient sector, the effectiveness of intensive care treatment was specifically assessed. This assessment was conducted using the following equation:


$$\Delta CFR_{\text{ICU}}=p_{\text{ICU}}\cdot \;\left(\mu -\alpha \right)$$ (1)

where $p_{\text{ICU}}$ is the probability of intensive care treatment, *μ* is the additional mortality rate of critical patients without ICU treatment, and *α* is the false positive rate of ICU admissions. As of May 7, 2021, marking the end of the third wave, approximately 3.1% of all positively tested cases received intensive care treatment [6] (see Table 1 for input values). Among those admitted to the ICU, approximately 29% died, according to the last report by the Robert Koch-Institut [6] stating the cumulative number of deaths in the ICU. This percentage was corroborated by an analysis of COVID-19 patients admitted to the ICU until December 31, 2021, which found a 33% mortality rate [18]. The counterfactual scenario assumed the absence of intensive care treatment, but not all patients in this scenario would necessarily succumb to the disease, given that that “good clinical practice demands that greater emphasis be placed on patient safety by limiting false negatives” [19]. The rate of false positive admissions to the ICU, that is, without medical indication, was set at 10% in the base case [9]. Similar to Cleary et al. [20], this base case assumes that 90% of critical patients would die without access to the ICU. In a sensitivity analysis, I considered the scenario where ICU patients receiving non-invasive ventilation (e.g., CPAP or BiPAP) or oxygen therapy are managed on general wards, resulting in a 40% to 50% higher mortality rate compared to ICU treatment. In this scenario, even with a 40% to 50% increase in mortality, the resulting rate remains below 90%.

**Table 1:** Input values and distributions used in the base case and sensitivity analysis.

Input	Mean (range)	Distribution	Reference
*Clinical and epidemiological data*			
Infection fatality rate	0.0083 (0.0069–0.0098)*	Beta (125, 14,914)	[7]
CFR in Germany	0.024	–	[6]
Probability of ICU indication	0.031	–	[6]
CFR in the ICU	0.29	–	[8]
False-positive ICU admissions	0.1 (0.1–0.25)	Beta (6, 54)	[9]
ICU cases	107,066	–	[6]
Hospital-related transmission of SARS-CoV-2 in hospitalized COVID-19 patients	0.059 (0.057–0.061)*	Beta (3,417, 54,159)	[10]
Infectiousness of asymptomatic individuals relative to symptomatic	0.75 (0.25–1.0)	Beta (3, 1)	[11]
Transmission rate reduction	0.10 (0.05–0.2)	Beta (5, 42)	[12,13]
COVID-19 outpatient cases	3,230,615		[6,14]
*Costs*			
ICU costs per admission	€36,661 (€21,475–€36,661)	Gamma (90, 409)	[15], Appendix
General ward costs for patients with an ICU indication	€4,140 (€3312–€4968)	Gamma (96, 43)	Appendix
General ward costs for patients with an outpatient indication	€2,095 (€1,676–€2,514)	Gamma (96, 12)	Appendix
Polymerase chain reaction test (publicly funded)	€43.56		[6]
Close contact evaluation and monitoring via telephone, minutes per case	30		[16]
Contact tracer hourly rate	€15		[17]

CFR, case fatality rate; ICU, intensive care unit.

*95% confidence intervals of the mean.

For the outpatient sector, a counterfactual scenario was designed in which all COVID-19 patients are treated as inpatients. To calculate the effectiveness of the outpatient sector, the following equation was used:


$$\Delta CFR_{\text{Outpatient}}=\left(\frac{\beta_{\text{Inpatient}}}{\gamma_{\text{Inpatient}}}-\beta_{\text{Inpatient}}\right)\;\cdot CFR$$ (2)

where $\beta_{\text{Inpatient}}$ is the hospital-related transmission rate and *γ* is the proportion of positivelytested individuals receiving inpatient care. The ratio $\frac{\beta_{\text{Inpatient}}}{\gamma_{\text{Inpatient}}}$ indicates the probability of hospital-acquired infections among those treated in the hospital. To determine the increase in CFR when all patients are treated as inpatients, I subtract the current hospital-related transmission rate.

Based on data from the Robert Koch-Institut [14], only 9% of all positively tested individuals as of May 7, 2021 [14] received inpatient care, meaning that 91% received outpatient care. It is known that inpatient treatment increases the probability of transmission [21]. During the outbreak in China in January 2020, 41.3% of hospitalized COVID-19 patients were reported to have acquired SARS-CoV-2 through hospital-related transmission [21]. Over time, however, the proportion of hospital-acquired infections decreased, indicating improved infection prevention measures or the effects of immunization, with rates lying at approximately 6% in Germany during the first three waves [10].

The calculation of the benefits of the outpatient sector is independent of the benefits of ICUs. The former focuses on the avoidance of a higher transmission rate if COVID-19 patients were treated as inpatients, whereas the latter centers on reducing the mortality of COVID-19 patients. It is worth noting that the outpatient sector did not directly prevent intensive care admissions. This would have required preventive measures with proven effectiveness, such as nirmatrelvir/ritonavir, which were not available during the first three waves.

For the local health departments, the counterfactual scenario involves the absence of contact tracking via telephone. However, this scenario only affects contacts of confirmed COVID-19 cases and not those of unreported ones. Unreported cases contribute to the infection’s spread even with local health departments’ presence. The calculation assumes conservatively that during the first wave, local health departments successfully followed up with all contacts of detected cases. This assumption is supported by self-reports from 152 of the 380 local health departments in Germany as of August 2020 [22].

To estimate the number of unreported COVID-19 cases, the infection fatality rate (IFR), which includes both reported and unreported cases, was taken into account. Dimpfl et al. [7] estimated the IFR of the Wuhan strain to be 0.83% in Germany, closely aligning with the World Health Organization’s [23] estimate of 0.75%. Although some studies indicate that hospitalization rates may have increased with the Alpha variant, the exact impact on death rates remains inconclusive. While undetected cases are likely to be asymptomatic or have mild symptoms, they can still be responsible for symptomatic cases in their contacts. Nevertheless, the infectiousness of asymptomatic cases seems to be somewhat lower. The U.S. Centers for Disease Control and Prevention [11] assumed an infectiousness of asymptomatic individuals relative to symptomatic individuals of 75% in the so-called “current best estimate.”

To calculate the impact of local health departments during the first wave, the following equation was used:


$$\Delta CFR_{\text{Health Departments}}=\frac{1}{\theta }\cdot \frac{1}{k}\cdot CFR$$ (3)

where *θ* is the unreported relative to the reported infection rate and ƙ is the infectiousness of asymptomatic individuals relative to symptomatic individuals.

Following up with all contacts becomes more difficult during larger outbreaks, regardless of the level of contact. As spread increased throughout November 2020, contact tracing became almost impossible. As of November 6, 2020, an estimated 75% of cases were not traceable in Germany [12].

The following formulas describe the theoretical relationship between the effectiveness of contact tracing and the resulting reduction in the reproduction number during the second and third waves:


$$R=E\cdot R_{\text{max}}$$ (4)

$$R_{\text{max}}=E_{\text{max}}\cdot R_{0}$$ (5)

where $R_{0}$ is the basic reproduction number, *E* is the effectiveness of contact tracing (expressed as a percentage), and *R* is the reduction in transmission due to contact tracing. These equations highlight that, theoretically, fully effective contact tracing $E=E_{\text{max}}$ can substantially reduce the reproduction number of COVID-19. Evidence suggests that under optimal conditions, contact tracing and subsequent isolation can reduce the effective reproduction number by approximately 40% $E_{\text{max}}$. This is based on models that consider the timely identification, isolation of contacts, and adherence to quarantine measures [13]. It is assumed that, ceteris paribus, the reduction in transmission leads to a proportional reduction in infections and deaths [13].

To calculate the aggregated impact of local health departments over the first three waves, a weighted average reduction of deaths was calculated, using the number of deaths in each period as weights.

### Cost-effectiveness

Secondary cost data, as detailed in Tables 1 and 2, were utilized to analyze cost-effectiveness. For costing services in both the inpatient and outpatient sectors, a top-down gross costing approach was applied. This method estimates input use in total (gross costing) rather than separately costing each input (e.g., labor hours) in these sectors. The gross costing is done top-down [25]. For contact tracing by the public health sector, a top-down micro-costing approach was used. Notably, the costs induced by preventing death (i.e., survival costs) were not included in isolating the costs of each healthcare sector.

**Table 2:** Outpatient costs associated with COVID-19 diagnosis and treatment according to the outpatient fee schedule of the statutory health insurance (as of Q2 2021) [24]. All costs are in euros.

Fee schedule item	Description	Fee
32816	Nucleic acid detection	39.40
12220	Physician laboratory service	1.56
40100	Transport of examination material and transmission of the examination result	2.60
03000	General practitioner flat rate	18.44^§^
13650	Additional flat rate for pulmonary diagnostic testing	34.60
13250	Additional flat rate for internal specialist treatment	16.80

^§^Average of age-specific remunerations.

ICU costs for treating COVID-19 patients were calculated based on an average patient trajectory, utilizing relevant diagnosis-related group (DRG) codes along with additional charges (“Zusatzentgelt”) on top of the DRG payments. Infrastructure costs were excluded since they are not funded by German payers. It should be noted that the DRG codes used were not specific to COVID-19 but rather covered ICU treatment with and without mechanical ventilation (see Appendix for detailed information). In a sensitivity analysis, the study applied a cost estimate of €21,475 as the average weighted cost, based on data published in news outlets for ICU patients with and without invasive ventilation [15].

The calculation of ICU costs involved subtracting the costs of comparator treatment, which refers to patients who were indicated for ICU care but who were instead treated in a general inpatient setting. For these patients, the assumption was made that, except for those falsely admitted to the ICU, they would have died without intensive care. The study applied a 10% rate for false positive ICU admissions, reflecting the proportion of patients who might have been admitted unnecessarily (see Table 1). This rate was used to adjust the costs of inpatient treatment outside the ICU. The cost calculation is summarized as follows:


$$\text{Δ}C_{\text{ICU}}=C_{\text{ICU}}-\alpha \cdot C_{\text{inpatient}}$$ (6)

where $\text{Δ}C_{\text{ICU}}$ is the incremental costs of ICU treatment compared to general inpatient treatment, $C_{\text{ICU}}$ is the average cost of treating a COVID-19 patient in the ICU, and $C_{\text{inpatient}}$ is the average cost of treating a COVID-19 patient in a general inpatient setting (non-ICU).

To assess the total ICU costs, the current study multiplied per-patient costs by the number of intensive care patients treated during the first three waves of the pandemic. This calculation is essential for making a meaningful comparison with the expenditures for the outpatient and local health department sectors.

To determine outpatient costs for treating COVID-19 patients, the study considered the detailed recommendations of the Schleswig-Holstein Association of Statutory Health Insurance Physicians [26,27] on outpatient management and outpatient billing of COVID-19 patients. Swab costs in the outpatient sector were excluded from the calculation since they are incurred independently of treatment setting and thus cancel out. Costs were calculated based on the outpatient fee schedule of the statutory health insurance in 2021 [24]. The specific fee items and costs are listed in Table 2.

The calculation of outpatient costs involved subtracting the costs of comparator treatment, which refers to patients who were instead treated in a general inpatient setting. For a detailed breakdown of the cost calculation, refer to the Appendix.

For assessing the cost-effectiveness of the local health departments, the study took into account labor costs related to investigating COVID-19 cases and tracing close contacts of confirmed cases. Additionally, the costs of polymerase chain reaction (PCR) testing for close contacts of COVID-19 cases were included, as these costs would not be incurred without contact tracing. To maintain compatibility, the study also incorporated testing costs in the ambulatory setting. Since the comparator was the absence of local health departments without a substitute, the costs attributed to local health departments were considered incremental.

The study determined the incremental cost-effectiveness ratios (ICERs) of the different healthcare sectors, each compared to the counterfactual scenario, using the following equation:


$$ICER=\frac{Cost\;of\;Healthcare\;Sector-Cost\;of\;Counterfactual}{Deaths\;Healthcare\;Sector-Deaths\;of\;Counterfactual}$$ (7)

Since the sectors were not mutually exclusive, the study did not determine dominance based on higher costs and fewer deaths avoided compared to another sector.

### Uncertainty analysis

The study performed one-way deterministic analyses to demonstrate the effects of the upper and lower bounds for each uncertain variable. This sensitivity analysis varied one parameter at a time, while keeping others constant, to evaluate its impact on the overall results. Complete national-level data from Germany, such as the CFR, probability of ICU admission, and CFR in the ICU, were not included in robustness testing. The ranges tested for robustness, shown along with their distributions in Table 1, were based on the referenced sources. Gamma distributions listed in Table 1 were defined using their shape and scale parameters. The results of the sensitivity analysis are presented using tornado diagrams.

## Results

### Effectiveness

If intensive care treatment had not been available for COVID-19 patients, it is estimated that all patients, except those falsely admitted without indication, would have succumbed to the disease. Consequently, intensive care medicine in Germany is assumed to have prevented a 1.9% increase in the CFR from COVID-19, which can be calculated as 3.1% multiplied by the difference between the additional fatality rate of critical patients without ICU treatment (71%) and the false positive rate (10%) (see Equation 1).

Regarding the effectiveness of the outpatient sector, the study explored the hypothetical scenario where all COVID-19 patients were treated as inpatients. In this counterfactual scenario, the COVID-19 case load would have increased by approximately 57% due to hospital-related transmissions $\left(\frac{6\%}{9\%}-6\text{\%}\right)\;$ (see Equation 2). This would lead to a correspondingrelative increase in the CFR by 57%, which translates to a 1.4% absolute increase in the CFR, based on the CFR of 2.4% as of May 7, 2021 [6].

To quantify the effectiveness of the local health departments during the first wave, the analysis took into account the presence of unreported COVID-19 cases and the relative infectiousness of asymptomatic individuals relative to symptomatic individuals. The calculation revealed that the unreported number was approximately 3.2 times larger than the total number of officially reported cases. By multiplying the reciprocal of the unreported rate by the reciprocal of the relative infectiousness, the study determined that local health departments had control over 46% of the death toll. Therefore, the absolute impact of local health departments is estimated to be 1.1% over the first wave (equal to 46% multiplied by the CFR of 2.4%, see Equation 3).

During the second and third waves, the transmission rate was reduced by 10%, given a maximum possible reduction of 40% with fully effective tracing and an actual tracing effectiveness of 25% (Equation 4). The weighted-average impact over the first three waves was calculated to be 14%, resulting in a reduction of the CFR by 0.3%.

### Cost-effectiveness

As indicated in Table 3, the base case analysis shows that hospitals played the most important role in avoiding deaths due to COVID-19, followed by the outpatient sector. Population spending on ICU care markedly surpasses that on outpatient care, approximately by a factor of 10.7. The outpatient sector is the most cost-effective option, as it dominates inpatient care for COVD-19 patients who can be treated in an outpatient setting. Compared to local health departments, it exhibits lower costs and higher effectiveness. However, it is worth noting that for the costs attributed to local health departments, only 17% is spent on contact tracing itself, while the majority of the expenses are allocated to PCR testing.

**Table 3:** Costs, effects, and cost-effectiveness of different health care sectors.

	Incremental spending	Incremental deaths avoided	Incremental costs per death avoided
Inpatient sector	€3,880,821,302	65,715	€59,055
Outpatient sector	−€6,402,177,007	48,340	−€132,440
Public health sector	€891,504,536	12,181	€73,188

### Sensitivity analysis

**Fig 1:**
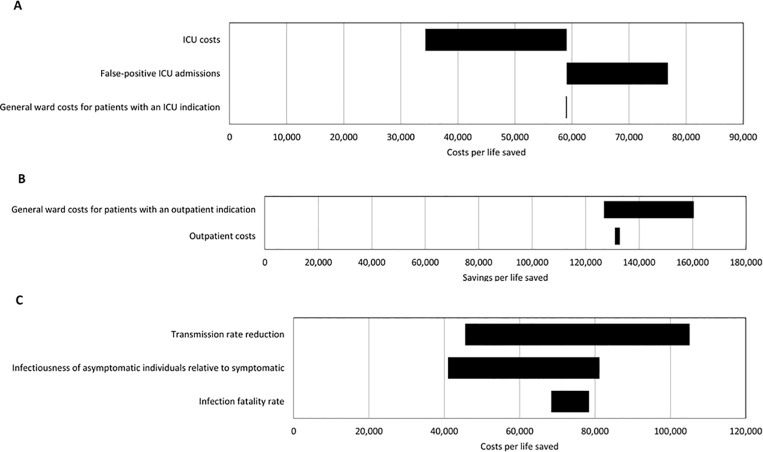
One-way sensitivity analysis on incremental cost-effectiveness ratios of different health care sectors. (A) Intensive care units (ICUs). (B) Outpatient sector. (C) Local health departments.

Fig 1 presents the quantitative results of the one-way sensitivity analysis on ICERs. It shows that the ICER for the public health sector varies significantly depending on the transmission rate reduction, with values ranging from €45,539 to €105,093. Additionally, the outpatient sector consistently generates cost savings. The sensitivity analysis thus reaffirms that the outpatient sector is consistently superior in cost-effectiveness compared to the other two sectors.

## Discussion

This study is the first of its kind to analyze the effectiveness and cost-effectiveness of different health care sectors in managing the first three waves of the COVID-19 pandemic. From an epidemiological perspective, the findings suggest that intensive care treatment, and therefore the inpatient sector, played a more important role in managing the first three waves in Germany, in preventing an increase in the CFR, compared to the outpatient sector and local health departments (1.9% versus 1.4% and 0.3% respectively). Including the contribution of inpatient non-intensive care would further support this finding. Thus, the current evidence does not support the notion that local health departments in Germany had a predominant impact during the first three waves, which is further reinforced by doubts about their ability to effectively follow up with all contacts of infected individuals [28].

The finding of this study aligns with the findings of a systematic review of empirical studies that examined the effectiveness of non-pharmaceutical interventions during the first wave of the pandemic, which did not find a clear association between contact tracing, quarantining, or isolation measures and COVID-19 deaths [29]. Similarly, a mathematical modeling study on the first wave in the U.S. suggested that investing significant resources in contact tracing beyond the baseline rate might not be cost-effective [30].

Despite these differences, it is important to recognize that the three sectors had different roles in managing COVID-19 and complement each other. The outpatient sector, local health departments, and inpatient care together formed a comprehensive system that addressed various aspects of the pandemic, from prevention and contact tracing to intensive medical treatment.

The study’s findings indicate that while the majority of COVID-19 patients in Germany received outpatient care, this does not necessarily imply that the outpatient sector had the greatest impact on reducing mortality. Nevertheless, the outpatient sector was demonstrated to be the most cost-effective option, as it dominated inpatient care. However, the ICERs for ICU care and local health departments were still considered acceptable, based on an opportunity cost threshold for cost-effectiveness analysis of approximately €90,000 per life year gained [31]. Based on an estimated gain of 2.1 life years per avoided death in the ICU [32] and 7.7 life years per avoided death in the general population [32], the ICERs for ICU care and local health departments were approximately €28,000 and €9,500 per life year gained, respectively. The cost-effectiveness of ICU care is further supported by a previous analysis showing that even for a low probability of utilization, expanding a staffed ICU bed reserve capacity remains cost-effective in Germany, considering the importance of being prepared for potential catastrophic scenarios [9].

The study’s limitations include reliance on secondary data, the absence of randomized controlled trials, and simplified counterfactual scenarios that may not fully capture the complexities of pandemic management. Additionally, the focus on direct medical costs excludes broader economic impacts, and assumptions made about mortality and transmission rates may not be universally applicable. One potential avenue for future research is to explicitly account for chains of infections and the dynamic behavior of COVID-19 spreading in cost-effectiveness analyses.

While the study did not analyze the impact of different healthcare sectors for each of the first three waves, general inferences can be made. The impact of the hospital sector may have increased in the third wave, as the Alpha variant, which was prevalent at that time, led to a higher probability of ICU admission without affecting ICU mortality [33]. At the same time, the impact of the outpatient sector may have diminished due to an increased risk of hospitalization associated with the Alpha variant compared to previous lineages [34]. Furthermore, as the Alpha variant was more transmissible [35], it also led to a significant number of asymptomatic cases, especially in settings with high vaccination rates, thus increasing the unreported infection rate and reducing the impact of contact tracing through local health departments.

This research has important implications for pandemic preparedness not only in Germany but also, for example, in low-resource settings. It suggests that when the goal is to minimize the number of deaths, it is crucial to expand ICU capacity, enhance staffing through recruitment and training, ensure a robust supply chain for critical medical supplies, and leverage advanced technology like tele-ICU and remote monitoring systems. When the goal is to maximize the number of life years gained, pandemic preparedness efforts should prioritize strengthening the role of the outpatient sector to prevent within-hospital transmissions resulting from unnecessary hospital admissions of infected patients. Additionally, improving the efficiency of contact tracing is crucial, especially when rapidly scaling up contact tracing during a pandemic. This could involve adopting digital tracing technologies and apps as a partial replacement for manual contact tracing via telephone. By understanding and implementing these measures, settings can enhance their ability to manage and respond to future pandemics more efficiently.

## Appendix

### Cost estimation for treating severe COVID-19 patients in intensive care units

The cost estimation for a typical severe COVID-19 patient admitted to an intensive care unit (ICU) was calculated based on diagnosis-related groups (DRGs) and additional charges (Zusatzentgelt) based on the 2022 German DRG catalogue. This estimation incorporates the probability of invasive mechanical ventilation, which was reported as 58% in the latest data from the Robert Koch Institute [8]. Given the likely decrease in this probability from March to May 2021, partly due to improved treatment protocols, a 55% probability was applied in the calculation.

For the purpose of this calculation, the DRGs specific to severe COVID-19 cases requiring prolonged mechanical ventilation, dialysis, and complex interventions were identified. These DRGs include, but are not limited to, A06A (prolonged ventilation >  1799 hours), A07A (ventilation >  999 hours), A13A (ventilation >  95 hours), and several other DRGs related to organ failure management and complex respiratory interventions (such as E40A and E36Z). Additionally, supplemental charges for specific interventions such as intermittent hemodialysis (ZE01.01), intermittent hemodiafiltration (ZE02), and extracorporeal photopheresis (ZE37) were considered.

The costs for each DRG were derived from their case mix index (CMI), which reflects the relative resource use associated with each category. The CMIs were multiplied by the base rate for 2021 (€3,747.98), as determined for the German healthcare system, to convert them into actual costs in euros. The probabilities of each DRG being billed for a typical COVID-19 ICU patient were estimated based on clinical patterns observed during the pandemic. These probabilities were then used to weight the individual DRG costs, yielding a more accurate reflection of the expected financial burden per patient.

For instance, A06A, representing the highest-cost cases involving ventilation for more than 1799 hours, was assigned a 5% probability, contributing significantly to the weighted average. DRGs such as A13A (ventilation >  95 hours) and E40A (ventilation >  24 hours) were considered to have higher probabilities of being billed (33% and 10%, respectively), reflecting the common clinical pathways for patients with severe respiratory distress due to COVID-19.

The total weighted DRG cost amounted to approximately €36,495. On top of this, the weighted cost of additional charges for treatments like dialysis and other interventions (€166) was included. The final estimated total cost for a typical severe COVID-19 ICU patient, encompassing both DRGs and additional charges, was approximately €36,661.

### Cost estimation for treating COVID-19 patients in a general inpatient setting without intensive care

The cost estimation for treating a COVID-19 patient as an inpatient, despite an indication for ICU care, was also calculated using DRGs and additional charges (Zusatzentgelt). This approach takes into account that the probability of ventilation on a general ward is generally lower than in the ICU, even for patients with an indication for ICU care. While the patient may have an indication for intensive care and ventilation, general wards typically lack the capacity to provide prolonged or invasive mechanical ventilation, which is a core function of ICUs. In general wards, patients may receive non-invasive ventilation (e.g., CPAP or BiPAP) or oxygen therapy, but the more invasive and resource-intensive forms of ventilation (such as mechanical ventilation with intubation) are almost exclusively managed in the ICU.

The DRGs selected for this calculation reflect common treatment pathways for non-ICU COVID-19 patients with severe symptoms. Key DRGs included E40C (respiratory conditions with ventilation >  24 hours, but without ICU-level complications), E79B (infections of the respiratory system without severe complications), L60C (kidney failure requiring dialysis without ICU-level care), and E65B (chronic obstructive pulmonary disease without ICU-level interventions).

The costs for each DRG were calculated based on their CMI, which reflects the relative resource consumption of the treatment. The CMIs were multiplied by the base rate for 2021 (€3,747.98) to determine the actual cost in euros. For example, E40C (ventilation >  24 hours without complications) has a CMI of 1.677, resulting in a cost of €6,284, while E79B (respiratory infections without complications) has a CMI of 0.747, leading to a cost of €2,801.

The probability of each DRG being billed was estimated based on typical clinical outcomes for non-ICU COVID-19 patients. For instance, E40C was assigned a 30% probability, reflecting the common use of non-invasive ventilation or brief ventilation periods in these cases. Other DRGs, like E79B and L60C, were assigned probabilities of 25% and 20%, respectively, based on their likelihood in non-ICU care. The weighted cost for each DRG was again calculated by multiplying the cost of the DRG (CMI ×  base rate) by its probability.

In addition to DRG costs, supplemental charges (Zusatzentgelt) for specific interventions were included. These additional charges covered treatments such as intermittent hemodialysis (ZE01.01), intermittent hemodiafiltration (ZE02), and extracorporeal photopheresis (ZE37), with corresponding probabilities based on the clinical treatment patterns observed for non-ICU COVID-19 patients.

The total weighted DRG cost for non-ICU COVID-19 patients was calculated to be approximately €4,096. When the weighted cost of additional charges (€44) was added, the total estimated cost for treating a COVID-19 patient in a general inpatient setting without ICU care was €4,140.

### Cost estimation for COVID-19 patients treated in inpatient instead of outpatient settings

The cost estimation for a COVID-19 patient treated in an inpatient setting despite an indication for outpatient treatment was derived based on relevant DRGs. These DRGs reflect the clinical conditions and resource usage associated with non-severe cases, including various degrees of respiratory insufficiency and respiratory infections.

To calculate the total cost, the CMI for each DRG was again multiplied by the base rate for 2021 in the German healthcare system (€3,747.98). DRGs associated with multi-day stays and varying levels of respiratory complications were considered, including E64C (respiratory insufficiency, multi-day stay, without very severe complications) with a CMI of 0.565 and E65C (chronic obstructive pulmonary disease, without very severe complications or complicating diagnoses, minimal treatment needs, multi-day stay) with a CMI of 0.545.

Single-day DRGs, such as E64D and E79C, were excluded, as they do not adequately reflect the typical inpatient cost for COVID-19 cases that are suitable for outpatient care but are nonetheless hospitalized. These cases generally require longer observation periods and multi-day stays due to ongoing monitoring and supportive care, which increases resource use beyond that of a single-day admission.

The probabilities of each DRG being billed for a typical COVID-19 inpatient were estimated based on observed clinical patterns. E64C was assigned a probability of 70%, representing the majority of mild COVID-19 cases requiring multi-day inpatient care for respiratory insufficiency. E65C was assigned a probability of 30%, covering cases with less intensive respiratory needs or mild chronic respiratory conditions, all managed without ICU-level care.

These probabilities were used to weight the individual DRG costs, providing a more accurate representation of the financial burden. After applying these probabilities and calculating the weighted costs for each DRG, the total estimated cost for treating a typical COVID-19 inpatient, who could potentially be treated in an outpatient setting, was approximately €2,095.
